# Classification of Ameloblastoma, Periapical Cyst, and Chronic Suppurative Osteomyelitis with Semi-Supervised Learning: The WaveletFusion-ViT Model Approach

**DOI:** 10.3390/bioengineering11060571

**Published:** 2024-06-05

**Authors:** Bohui Liang, Hongna Qin, Xiaolin Nong, Xuejun Zhang

**Affiliations:** 1 School of Computer, Electronics and Information, Guangxi University, Nanning 530004, China; hliangcs@foxmail.com; 2 School of Information and Management, Guangxi Medical University, Nanning 530021, China; qhngxmu@foxmail.com; 3 College & Hospital of Stomatology, Guangxi Medical University, Nanning 530021, China

**Keywords:** CBCT panoramic images, semi-supervised learning, medical image classification, computer-aided diagnosis

## Abstract

Ameloblastoma (AM), periapical cyst (PC), and chronic suppurative osteomyelitis (CSO) are prevalent maxillofacial diseases with similar imaging characteristics but different treatments, thus making preoperative differential diagnosis crucial. Existing deep learning methods for diagnosis often require manual delineation in tagging the regions of interest (ROIs), which triggers some challenges in practical application. We propose a new model of Wavelet Extraction and Fusion Module with Vision Transformer (WaveletFusion-ViT) for automatic diagnosis using CBCT panoramic images. In this study, 539 samples containing healthy (*n* = 154), AM (*n* = 181), PC (*n* = 102), and CSO (*n* = 102) were acquired by CBCT for classification, with an additional 2000 healthy samples for pre-training the domain-adaptive network (DAN). The WaveletFusion-ViT model was initialized with pre-trained weights obtained from the DAN and further trained using semi-supervised learning (SSL) methods. After five-fold cross-validation, the model achieved average sensitivity, specificity, accuracy, and AUC scores of 79.60%, 94.48%, 91.47%, and 0.942, respectively. Remarkably, our method achieved 91.47% accuracy using less than 20% labeled samples, surpassing the fully supervised approach’s accuracy of 89.05%. Despite these promising results, this study’s limitations include a low number of CSO cases and a relatively lower accuracy for this condition, which should be addressed in future research. This research is regarded as an innovative approach as it deviates from the fully supervised learning paradigm typically employed in previous studies. The WaveletFusion-ViT model effectively combines SSL methods to effectively diagnose three types of CBCT panoramic images using only a small portion of labeled data.

## 1. Introduction

Ameloblastoma (AM), periapical cyst (PC), and chronic suppurative osteomyelitis (CSO) represent three distinct diseases within the maxillofacial region. Diagnosis of these conditions typically relies on a combination of imaging techniques, clinical pathology, and detailed histological analysis [1]. This complexity demands that doctors not only possess a wealth of professional knowledge and experience but also maintain a discerning perspective to guarantee diagnostic precision and devise effective treatment strategies.

**Figure 1:**
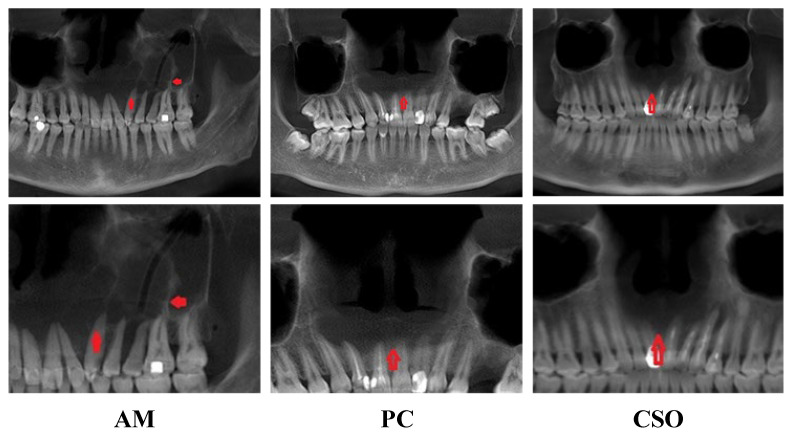
CBCT panoramic images of AM, PC, and CSO. The red arrows in the image indicate the lesion areas.

In terms of clinical pathology, AM frequently occurs in the mandible [2], CSO is more common in the ascending ramus and body of the mandible [3], and PC typically occurs in the soft and hard tissues around the root apex [4]. The imaging features of these three diseases all show low-density bone destruction. The lesion margins of AM and PC are clearer than those of CSO, which is a distinguishing point in traditional imaging. Figure 1 depicts the CBCT panoramic images of AM, PC, and CSO.

Although these conditions share some overlapping imaging features, they also exhibit distinctive characteristics that aid in their differentiation. AM is characterized by root resorption and displacement of adjacent teeth, often accompanied by jaw expansion, and typically appears on imaging as a multicystic lesion with well-defined borders. PC occurs in the alveolar bone around the apex of the tooth roots and may invade soft tissues after eroding the cortical plates. A hallmark of PC is the presence of a non-vital pulp, which may not be immediately apparent during radiographic diagnosis. Radiographically, it presents as a round or oval radiolucent area with well-defined borders and may be associated with cortical plate erosion. However, PC is not frequently diagnosed based on radiographs alone, as a lesion must exceed 1.5 to 2.0 cm in diameter before a reliable diagnosis can be made [5]. CSO presents differently from both AM and PC. While AM is generally well-defined and localized, and PC is specifically located at the tooth root apex, CSO can manifest with varying degrees of severity. Radiographically, CSO shows bone destruction, medullary cavity expansion, periosteal reaction, and mixed areas of sclerosis and radiolucency, with larger lesions (greater than 4 cm in diameter) disrupting surrounding anatomical structures and complicating the differential diagnosis [6].

While trained and experienced diagnosticians can often identify these features, the process can be time-consuming and requires significant training and experience. This challenge is exacerbated when clinicians are too busy to thoroughly examine and diagnose the images, potentially impacting the accuracy of their diagnoses [7]. This is where artificial intelligence (AI) can play a significant role. AI systems are designed to analyze large volumes of imaging data, identify subtle patterns, and potentially reduce the subjectivity and variability associated with human judgment. Moreover, AI programs can provide reliable diagnostic support to less experienced doctors, especially when these programs are trained on diagnostic results from domain experts. This can offer a potentially reliable diagnostic option for patients in underdeveloped regions.

In recent years, the increasing application of AI in oral and maxillofacial imaging has greatly facilitated the early diagnosis, accurate prognostic prediction, and effective treatment planning for maxillofacial diseases [8,9], with a particular focus on X-ray and cone-beam computed tomography (CBCT) applications [10,11,12,13]. Commonly, routine dental radiography can often accidentally reveal jaw lesions, and, for dental practitioners, it is challenging to distinguish them on radiological images [14]. In order to achieve more accurate and timely diagnoses of jaw diseases, some studies have utilized diagnosis models based on deep learning to distinguish AM, Odontogenic Keratocyst (OKC), PC, and other diseases in panoramic X-ray and CBCT images and have achieved encouraging progress [15,16,17]. CBCT panoramic images can generate intuitive panoramic views of the oral cavity, providing more comprehensive information and more accurate diagnostic values for doctors. Although several studies have proposed reconstruction methods for CBCT panoramic images [18,19], research on employing deep learning models to classify these reconstructed CBCT panoramic images remains notably scarce. In addition, since the amount of data on jaw lesions is usually small, and previous studies on jaw disease classification have focused on fully supervised learning using a limited number of jaw lesion images, this is prone to model overfitting problems and insufficient generalization of the model. Semi-supervised learning (SSL) offers significant advantages for jawbone lesion image classification. By leveraging both labeled and unlabeled data, it enhances model performance and generalization while minimizing the need for manual annotation and improving efficiency. This novel approach provides a solution to the challenges in medical imaging classification of jawbone lesions, with the potential to enhance clinical diagnosis and treatment. Through semi-supervised learning, we can harness the full potential of medical imaging data, improving the accuracy and reliability of lesion classification and providing clinicians with a more robust decision-making foundation.

Based on the aforementioned reasons, this study aims to develop an innovative deep learning model, WaveletFusion-ViT, which leverages SSL to automatically classify typical and common maxillofacial diseases, AM, PC, and CSO, using a limited set of labeled CBCT panoramic images.

## 2. Materials and Methods

This study design was reviewed and confirmed by the Ethics Committee of Guangxi Medical University (No. 2022-0186). All the procedures were carried out in strict accordance with the prescribed rules and regulations.

### 2.1. Patient and Data Collection

The original data of the present study were sourced from the Hospital of Stomatology, Guangxi Medical University between January 2018 and December 2023 for the present experiment. All the CBCT panoramic images were captured with an i-CAT™ 17-19 (Imaging Sciences International, Hatfield, PA, USA) and exported to JPG format using the i-CATVision™ software (version 1.9) with the Tru-Pan feature. Based on the histopathological diagnoses by pathologists from the Guangxi Medical University College of Stomatology, we collected 154 CBCT panoramic images of healthy individuals and 385 CBCT panoramic images of lesion samples. The lesion samples, which were all confirmed by histopathological examination, included 181 ameloblastoma (AME) samples, 102 chronic suppurative osteomyelitis (CSO) samples, and 102 periapical cysts (PC) samples. Even if confirmed on clinical records, participants were excluded if they (1) had images with apparent artifacts involving the regions of interest (ROI), (2) had images with severe distortion or artificial noise, or (3) were follow-up patients. And, if a sample met the following criteria, it was defined as a healthy sample: (1) Clinical data and imaging did not reveal any pathological jawbone diseases. (2) It was confirmed by an experienced physician that no lesions were detected from the collected images. A panel of three experienced oral radiologists independently reviewed the dataset of radiographs, and their consensus diagnosis was used as the human diagnostic ‘gold standard’.

**Figure 2:**
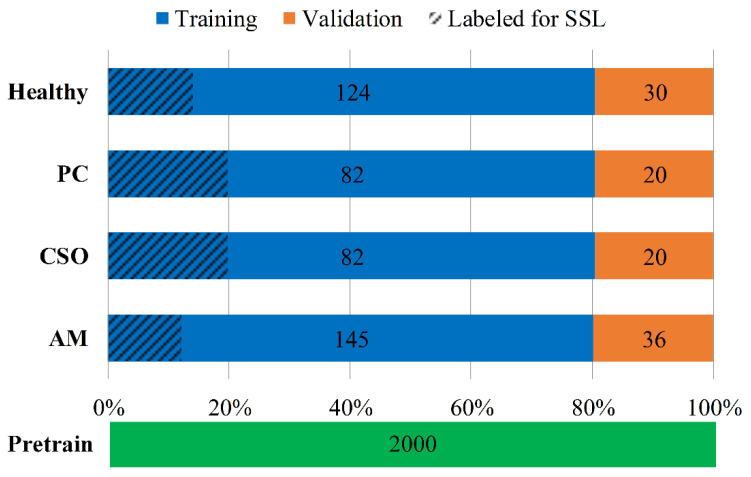
The dataset statistic of CBCT panoramic images.

In this experiment, 80% and 20% samples were used for training and validation in each cross-validation fold, respectively. In addition, 2000 panoramic radiographs of healthy individuals from a previously established dataset [20] were used for pre-training the network. Finally, a pre-train dataset with 2000 healthy samples’ panoramic radiographs and an equalized classification dataset with 539 samples’ CBCT panoramic images were included in this study. Figure 2 summarized more details about the collected dataset.

### 2.2. Image Preprocessing and Augmentation

The size of the original CBCT panoramic radiograph is about 1200 × 450 pixels, which is too large for normal deep neural networks (DNNs). In this experiment, we resized the images to a more manageable 224 × 224 pixels. Subsequently, we employed a suite of data augmentation techniques to enrich our dataset and prevent overfitting. These techniques included horizontal flipping, random rotations, random cropping, and histogram equalization, each contributing to a more robust and varied dataset. During training, each epoch had a 50% chance of applying these data augmentation strategies to a given image, thereby introducing a diverse range of variations. This probabilistic approach ensured that our model was exposed to a broad spectrum of data scenarios, simulating real-world conditions. After 200 epochs of training, this method yielded up to 43,100 new augmented images (431 × 200 × 0.5) within each cross-validation fold, significantly expanding our original dataset and enhancing the model’s ability to generalize from the training data to unseen cases.

### 2.3. Proposed Framework

**Figure 3:**
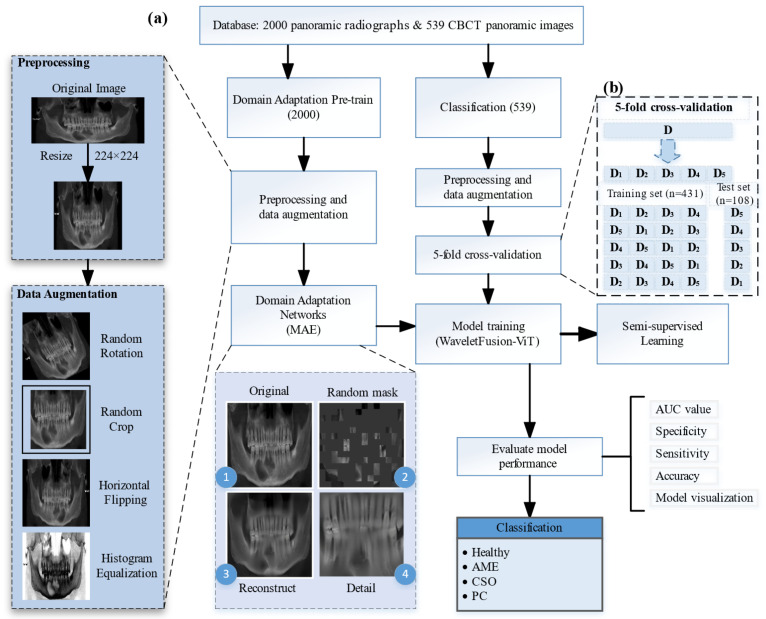
Proposed framework. (**a**) A flow chart of the deep learning model building process. (**b**) A schematic diagram of five-fold cross-validation. The meaningful representation of the medical images is used by replacing the encoder of the WaveletFusion-ViT network with the pre-trained domain adaptation network encoder.

The entire framework consisted of three parts: (1) a semi-supervised network, (2) domain adaptation networks, and (3) a Wavelet Extraction and Fusion Module. An overview of the proposed framework is represented in Figure 3.

#### 2.3.1. Semi-Supervised Learning

**Figure 4:**
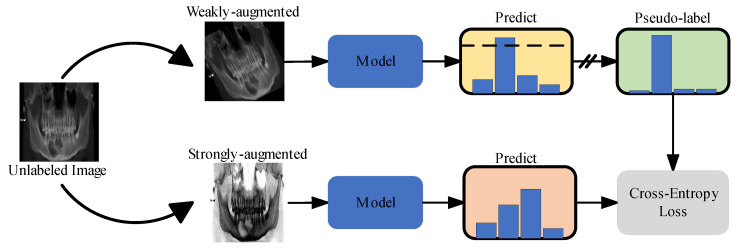
Illustration of semi-supervised learning (SSL).

The excellent performance of DNNs largely depends on supervised learning with sufficient labeled data. In the realm of medical image analysis, the lack of large training datasets, data annotation issues, and data imbalances represent the three main challenges faced by DNNs [21,22]. Unlike supervised learning (SL), which can learn high-dimensional features from labeled data, semi-supervised learning (SSL) combines labeled and unlabeled data during model training. This means that, compared to SL, using SSL algorithms allows the model to learn more meaningful representations from smaller datasets, which is helpful when lacking a mass of labeled medical images data. In recent years, with a surge of interest in SSL, many applications of SSL in medical image processing are emerging [23,24,25]. An increasing number of works have reported that SSL approaches generally perform better than high quality supervised baselines [26,27]. In this study, we attempted to improve the model performance for maxillofacial diseases images classification by utilizing a small amount of labeled data and a large amount of unlabeled data. The FreeMatch [28] algorithm was selected as the SSL framework for this experiment. The workflow of the SSL framework we used is shown in Figure 4. The labeled and unlabeled data are denoted as $D_{L}=\{x_{i}^{l}{\}}_{i=1}^{N_{L}}$ and $D_{u}=\{x_{i}^{u}{\}}_{i=1}^{N_{u}}$, respectively, where $x_{i}^{l}$ and ${\;x}_{i}^{u}$ were the labeled and unlabeled training images. $N_{L}$ and $N_{u}$ represented the number of training samples in $D_{L}$ and $D_{u}$, respectively. During a training batch, for data in $D_{L}$, the algorithm first calculated the supervised cross-entropy (CE) loss $L_{s}$. And, for unlabeled images, weak and strong augmentation were applied to the same image, respectively, and then the predicted category of the former was taken to generate a pseudo-label and calculate the CE loss $L_{u}$ for the two images. Finally, the model was optimized using a joint objective ${L=L}_{s}+L_{u}$. During each cross-validation process, we randomly selected 25 samples from each category in the training set, totaling 100 labeled samples for the SSL training (Figure 2).

#### 2.3.2. Domain Adaptation Networks

When we directly transferred the deep learning state-of-the-art (SOTA) model pre-trained on the ImageNet dataset to medical images, the performances were often not as expected. One important reason was that the distribution of natural images and medical images were different, which was referred to as the “domain shift” problem [29]. As a promising solution to tackle domain shift among medical image datasets, domain adaptation has attracted increasing attention in the field [30]. Inspired by the above facts, we proposed a domain adaptation network based on self-supervised learning to diagnose jaw cysts through a large number of healthy samples. In this experiment, the domain adaptation network we adopted was masked autoencoders (MAE) [31]. It worked in the following way: masking random patches of the input image and then training the model to reconstruct the missing pixels. Through this approach, the domain adaptation networks could learn the meaningful representation of medical images from a large number of healthy samples, which enabled the alleviation of the domain shift problem to a certain extent.

#### 2.3.3. Wavelet Extraction and Fusion Module

The rich texture information contained in medical images is crucial for the correct diagnosis of diseases. In recent years, a series of machine-learning-based radiomics studies on jawbone pathologies have shown that texture features were the most commonly used feature quantity, and texture analysis showed potential to contribute to radiologists’ reports [32]. However, the repeated up-sampling and down-sampling in the encoder-decoder structure of DNNs lead to the loss of texture details, seriously affecting the classification performance of the model. Therefore, we proposed a wavelet extraction and fusion module (WEFM), which could effectively extract and restore the high-frequency texture details of images by utilizing the frequency domain characteristics of the wavelet transform, providing additional information for spatial domain feature fusion.

**Figure 5:**
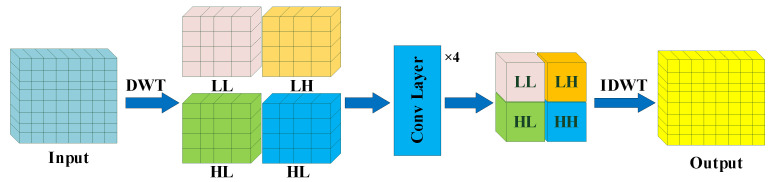
The architecture of our proposed wavelet extraction and fusion module (WEFM). We extracted four frequency sub-bands, LL, LH, HL, and HH, from input features by the wavelet transforms. Then, the frequency sub-bands were fused by three-layer convolution. Finally, we obtained a final output via the wavelet inverse transform.

The structure of the WEFM is shown in Figure 5. First, we divided the input $X_{input}$ into four different frequency sub-bands via the wavelet transform, which represented the three classification labels for the skeletal classification. These frequency sub-bands could be defined as follows:

(1) $$\left\{X_{LL},\;\;X_{LH},\;\;X_{HL},\;\;X_{HH}\}=DWT(X_{input}\right)$$

where DWT(·) denotes the operation of the wavelet transform. $X_{LL}$, $X_{LH}$, $X_{HL}$ and $X_{HH}$ denote the feature of four frequency sub-bands, respectively. In order to fully fuse the information of these sub-bands, each of them is input into four-layer convolutional neural networks (CNNs). This can be described as follows:

(2) $$X_{i\_t,\;\;i\in \{LL,\;LH,\;HL,HH\}}=F_{conv4}(X_{i})$$

where $F_{conv4}(\cdot )$ denotes the four-layer CNNs, $X_{i\_t,\;i\in \{LL,\;LH,\;HL,HH\}}$ represent the four frequency sub-band features fused by the four-layer CNNs. We finally obtain the output result $X_{out}$ through the wavelet inverse transform. It can be formulated as:

(3) $$X_{out}=IDWT(X_{i\_t,\;\;i\in \{LL,\;LH,\;HL,HH\}})$$

where IDWT(·) denotes the operation of the wavelet inverse transform.

**Figure 6:**
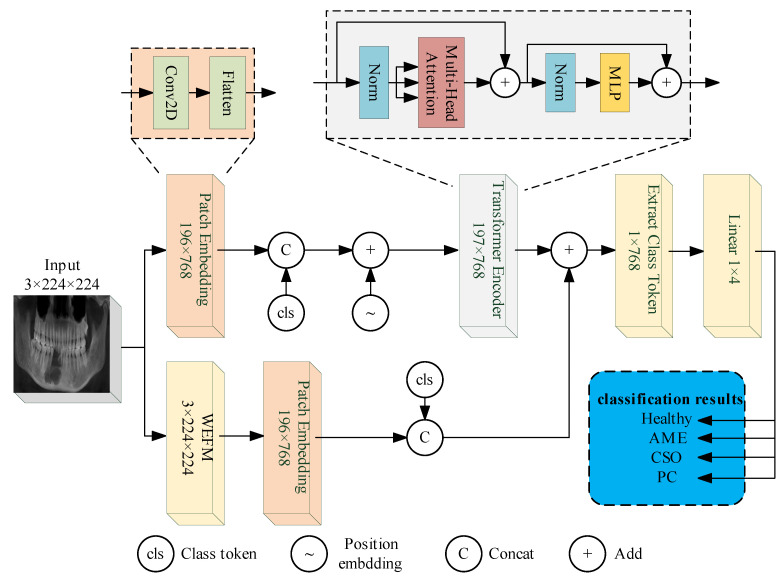
The architecture of proposed WaveletFusion-ViT model.

Finally, we combined the WEFM module with the Vision Transformer (ViT) [33], called WaveletFusion-ViT, as the classification model for this experiment. The schematic representation of the proposed model is depicted in Figure 6. Furthermore, we enhanced the model’s ability to capture meaningful texture features from medical images by substituting the WaveletFusion-ViT network’s encoder with an encoder from a pre-trained domain adaptation network.

### 2.4. Training Setup

The network architecture was built via Pytorch deep learning framework (version 2.1.2). The programming language was Python, and the operating system was Ubuntu 22.04 LTS. The model in this study was trained with 200 epochs using the AdamW optimizer and cross-entropy loss function after the pre-trained model’s parameters were initialized. All training procedures in this study were performed on a computer equipped with an NVIDIA GeForce RTX 4090 GPU (NVIDIA Corporation, Santa Clara, CA, USA).

### 2.5. Model Assessment

The following classification metrics were used to assess the performance of the classification network: Sensitivity (SN), Specificity (SP), Classification Accuracy (ACC).

The model was evaluated using five-fold cross-validation, where the dataset used was randomly divided into five parts based on the proportion of label categories. The means and standard deviations of the evaluation metrics were reported.

We created a Gradient-weighted Class Activation Mapping (Grad-CAM) to better comprehend the learning styles of the model [34]. The Grad-CAM visually highlighted the areas of the CBCT panoramic images that were most informative in terms of distinguishing between maxillofacial diseases classifications. In addition, confusion matrices, Receiver Operating Characteristic (ROC) curves, and the value of area under the ROC curve (AUC) were acquired to evaluate the classification performance.

## 3. Results

### 3.1. Sample Characteristics

The dataset’s baseline details are presented in Table 1. The average ages for AME, CSO, PC, and Healthy cases were 33.81 ± 15.82, 44.23 ± 21.98, 34.92 ± 16.05, and 28.20 ± 10.97 years (mean ± standard deviation), respectively. A predilection for the mandible was observed in AME, CSO, and PC cases. A statistically significant correlation (*p* < 0.001) was found between gender, age, and the location of jawbone lesions across the three diseases. Additionally, a notable statistical significance existed between the gender, age, and cohort of healthy individuals.

### 3.2. Model Classification Performance

**Figure 7:**
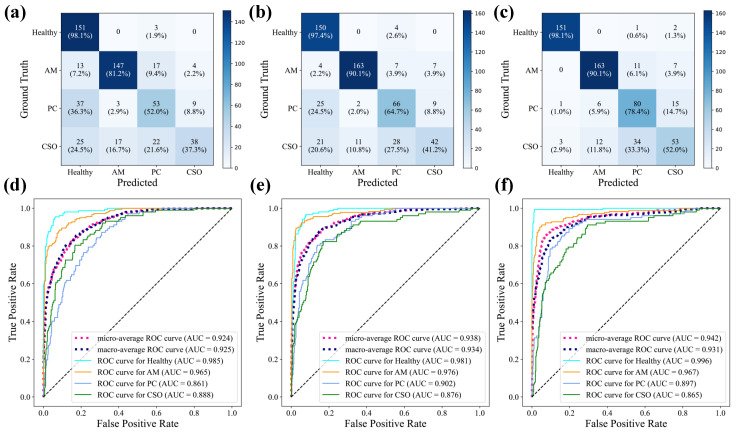
Multiclass confusion matrix of (**a**) fully supervised Densenet-121 model, (**b**) fully supervised ViT-B/16 model, and (**c**) our proposed method. Receiver operating characteristic curves of (**d**) fully supervised Densenet-121 model, (**e**) fully supervised ViT-B/16 model, and (**f**) our proposed method.

Figure 7 shows the confusion matrix for the classification of AM, PCs, CSO, and healthy jaws using either WaveletFusion-ViT, ViT-B/16, or Densenet-121. Table 2 summarized the classification results of sensitivity, specificity, and accuracy for each disease. The proposed method’s average sensitivity, specificity, and accuracy score of our study patients were 79.60%, 94.48%, and 91.47%, respectively. For comparison, we performed a five-fold cross-validation with a fully supervised approach on the same dataset, with results also depicted in Table 2. Our methodology demonstrated superior classification efficacy compared to the fully supervised approach.

**Figure 8:**
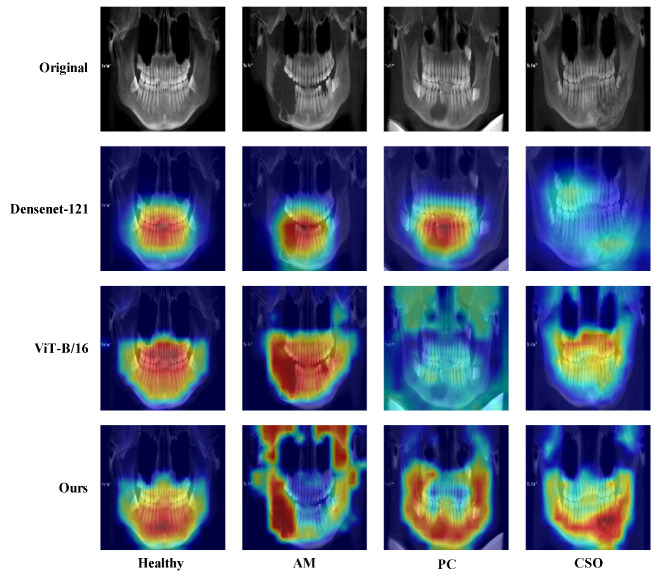
Grad-CAM visualization of images in four-class classifications. Red represents high attention, and blue means low attention.

Additionally, Figure 7 illustrates the ROC curves and AUC scores for the four-class categorization, revealing AUC scores for AM, PC, CSO, and healthy samples at 0.967, 0.897, 0.865, and 0.996, respectively, using our proposed method. Grad-CAM analysis, shown in Figure 8, highlighted the critical regions for classification within the upper and lower jaw areas. Direct visual analysis suggested that the primary activation zones in the classification network were situated both peripherally and centrally to the lesion, implying an absence of overfitting within the trained neural network.

**Figure 9:**
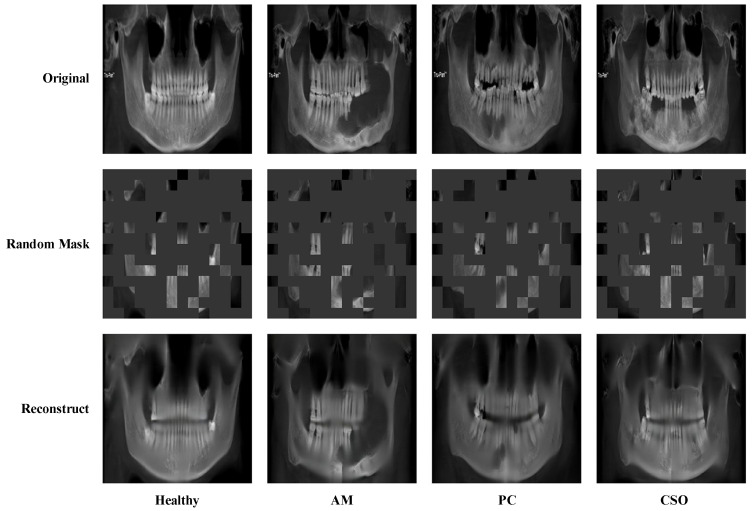
Visualization of the domain adaptation pre-train process. The original image is randomly masked by 75% and then reconstructed using the domain adaptation network.

The domain adaptation network pre-train process is depicted in Figure 9. Seventy-five percent of the original image was randomly masked and then input into a reconstruction network to rebuild the original image. The reconstructed image closely mirrored the original, preserving essential texture details.

## 4. Discussion

While oral surgeons frequently utilize panoramic X-rays for diagnostic purposes, research indicates that their capacity to identify lesions within these X-rays can be constrained [17]. Deep learning technology offers a spectrum of applications for analyzing oral and maxillofacial medical images, encompassing tasks such as classification [8], object detection [35], and segmentation [36]. Yet, the utilization of deep learning for CBCT panoramic images to diagnose maxillofacial diseases remains sparsely documented, which could be attributed to the nascent stage of deep learning applications in panoramic image reconstruction [19], with the associated techniques and methodologies still evolving.

The application of deep learning technology in medical imaging can assist busy or inexperienced oral surgeons in detecting and diagnosing oral diseases more accurately, thereby enhancing treatment outcomes and patient prognosis. The use of automated diagnostics based on deep learning as a clinical aid for diagnosing various diseases has gained widespread attention. However, there has been no reported research on applying semi-supervised learning to the automatic classification models for jawbone diseases. For the automatic categorization application of the diagnostic classification of maxillofacial diseases, we developed a representative DNN model, WaveletFusion-ViT, by utilizing 539 CBCT panoramic images. A novel Wavelet Extraction and Fusion Module (WEFM) was designed to extract the high-frequency texture details of images, in order to improve the performance of the model in disease classification. It was evident that the WaveletFusion-ViT model can accurately classify maxillofacial diseases, with >91% accuracy and >0.93 AUC after conducting a five-fold cross-validation on the classification dataset.

In contrast to the 89.05% accuracy achieved by fully supervised methods, our approach attained an impressive 91.47% accuracy using less than 20% labeled samples, marking a significant improvement. The categories of healthy individuals, AMs, and PCs exhibited heightened classification sensitivity, indicating that these lesions have distinctive radiological characteristics that our model may more readily identify. Conversely, the classification sensitivity for CSO was lower than that for other lesions (shown in Table 2), likely due to its heterogeneous radiographic manifestations that complicate accurate assessment, which is corroborated with the same research findings of Fullmer et al. [37]. The ability to accurately distinguish CSO from other conditions in radiological imaging holds substantial clinical value, given the potential overlap in radiographic features [38,39]. Previous studies typically involved manually segmenting the region of interest (ROI) from two-dimensional CBCT slices before using them as input for deep learning model. In contrast, our method allows for the direct use of the entire CBCT panoramic image obtained from imaging equipment as input, significantly reducing the complexity of practical clinical applications. Moreover, even without the prior knowledge of manually delineated ROI and in the presence of potentially confusing cases such as CSO, our approach still achieved a classification accuracy of 93.32% for AM with a limited number of labeled samples, exceeding the result of 84.6% by Chai et al. [15] and 91.37% by Bispo et al. [16]. This indicates that the model proposed in our study has a high capability for learning and generalizing disease features. In the field of medical imaging, acquiring annotated images often comes with high costs. By employing semi-supervised learning methods, we can make more efficient use of unlabeled data, thereby enhancing the model’s performance. Furthermore, the current research has not yet explored the application of deep learning techniques in CSO, marking our study as pioneering in a new field.

Deep learning is often perceived as a ‘black box’, posing a significant challenge in the medical field where doctors and patients require an understanding of the basis and reasoning behind predictive results to make accurate diagnostic and treatment decisions [40]. The usage of Grad-CAM has increased the reliability and interpretability of our method. Figure 8 provides a visual representation of the Grad-CAM results, with red areas indicating the regions of the image the model focuses on when generating classification results, offering crucial insights for medical professionals.

This study also has certain limitations. Firstly, all the image data originated from a single hospital and were captured by the same equipment, which may limit the broad applicability of the findings. Secondly, the relatively small sample sizes of CSO and PC results in the lower classification sensitivity observed. Additionally, it is important to note that, while our study focused on chronic suppurative osteomyelitis (CSO), it is not the only form of osteomyelitis. Drug-related osteomyelitis (DRO), for example, represents another significant subset of cases, accounting for approximately 12 out of the 102 CSO cases in our dataset. However, due to the relatively small number of DRO cases, we did not provide a separate analysis for this subtype in our study. Future research endeavors could explore the inclusion of DRO in the classification model, as its distinct clinical features and etiology may require specific diagnostic considerations. Furthermore, the current research method has not yet been validated for other types of jaw disease classification.

To enhance the universal applicability of the research findings, future work could involve collecting images from various hospitals taken with different equipment. This would help build a more representative dataset and improve the model’s generalization capabilities. To address the issue of insufficient case numbers, future research should seek more collaborative opportunities to enrich the sample size and could employ data augmentation strategies to increase the diversity of the training set, thereby improving the model’s accuracy in identifying these diseases. Moreover, exploring the application of this method in a broader range of disease classification tasks represents an important direction for future research.

## 5. Conclusions

In this research, we introduce an innovative semi-supervised learning-based approach for the automatic classification of maxillofacial diseases. Firstly, we constructed a novel DNN model WaveletFusion-ViT, which utilizes wavelet extraction and fusion modules to extract and learn high-frequency texture features from images, in order to improve the performance of the model. Secondly, in order to address the domain transfer problem from natural images to medical images, a domain adaptive network using the MAE method was applied to self-supervised training, and the resulting trained weights were transferred into the WaveletFusion-ViT model. Finally, the model underwent training through an SSL process and evaluation by five-fold cross-validation. The results of our investigation preliminarily indicate that our method may have an advantage in the precise classification of maxillofacial diseases with a limited set of labeled samples, potentially surpassing the capabilities of traditional fully supervised learning models. These findings provide initial evidence of the effectiveness of our approach; however, further research and validation are required to confirm its reliability.

## Figures and Tables

**Table 1:** Baseline characteristics of patients diagnosed with AME, CSO, PC, and healthy individuals.

Characteristics		AME (*n* = 181)	CSOJ (*n* = 102)	PC (*n* = 102)	Healthy (*n* = 154)	*p*-Value
Gender						<0.001
	Male	114 (63%)	58 (56.9%)	38 (37.3%)	35 (22.7%)	
	Female	67 (37%)	44 (43.1%)	64 (62.7%)	119 (77.3%)	
Age		33.81 ± 15.82	44.23 ± 21.98	34.92 ± 16.05	28.20 ± 10.97	<0.001
Location						<0.001
	Maxilla	8 (4.4%)	13 (12.7%)	62 (60.8%)	/	
	Mandible	173 (95.6%)	89 (87.3%)	40 (39.2%)	/	

**Table 2:** Performance of four-class classifications for healthy, AM, PC, and CSO samples.

Method Type	Method	Category	Sensitivity (%)	Specificity (%)	Accuracy (%)
Fully- Supervised	Densenet-121	Healthy	98.06 ± 3.87	80.52 ± 9.03	85.52 ± 5.52
		AM	81.22 ± 3.67	94.42 ± 1.95	89.98 ± 0.67
		PC	52.24 ± 16.3	90.40 ± 6.32	83.12 ± 3.36
		CSO	36.95 ± 12.73	97.02 ± 2.01	85.72 ± 1.71
		Means	67.12 ± 5.25	90.59 ± 1.39	86.09 ± 1.77
	ViT-B/16	Healthy	97.35 ± 3.88	87.07 ± 8.37	89.98 ± 5.95
		AM	90.08 ± 5.67	96.38 ± 3.35	94.25 ± 1.23
		PC	64.76 ± 13.92	91.07 ± 4.56	86.08 ± 3.08
		CSO	41.10 ± 8.67	96.33 ± 0.87	85.90 ± 0.59
		Means	73.32 ± 4.35	92.70 ± 1.37	89.05 ± 2.13
Semi- Supervised	WaveletFusion-ViT	Healthy	98.04 ± 2.59	98.96 ± 1.52	98.70 ± 1.11
		AM	90.06 ± 5.14	94.98 ± 3.78	93.32 ± 1.59
		PC	78.33 ± 16.23	89.47 ± 4.90	87.39 ± 3.22
		CSO	51.95 ± 7.91	94.51 ± 3.11	86.46 ± 1.60
		Means	79.60 ± 2.74	94.48 ± 0.70	91.47 ± 1.11

## Data Availability

Datasets access is available from the corresponding author upon reasonable request. We will provide the data only after completing the necessary legal procedures within China and exclusively for the purpose of re-evaluating the scientific credibility.
